# Smartphone three-dimensional imaging for body composition assessment using non-rigid avatar reconstruction

**DOI:** 10.3389/fmed.2024.1485450

**Published:** 2024-10-07

**Authors:** Grant M. Tinsley, Christian Rodriguez, Christine M. Florez, Madelin R. Siedler, Ethan Tinoco, Cassidy McCarthy, Steven B. Heymsfield

**Affiliations:** ^1^Energy Balance & Body Composition Laboratory, Department of Kinesiology and Sport Management, Texas Tech University, Lubbock, TX, United States; ^2^Pennington Biomedical Research Center, Louisiana State University System, Baton Rouge, LA, United States

**Keywords:** 3D scanning, body fat, smartphone, optical imaging, digital anthropometry

## Abstract

**Background:**

Modern digital anthropometry applications utilize smartphone cameras to rapidly construct three-dimensional humanoid avatars, quantify relevant anthropometric variables, and estimate body composition.

**Methods:**

In the present study, 131 participants ([73 M, 58 F] age 33.7 ± 16.0 y; BMI 27.3 ± 5.9 kg/m^2^, body fat 29.9 ± 9.9%) had their body composition assessed using dual-energy X-ray absorptiometry (DXA) and a smartphone 3D scanning application using non-rigid avatar reconstruction. The performance of two new body fat % estimation equations was evaluated through reliability and validity statistics, Bland–Altman analysis, and equivalence testing.

**Results:**

In the reliability analysis, the technical error of the measurement and intraclass correlation coefficient were 0.5–0.7% and 0.996–0.997, respectively. Both estimation equations demonstrated statistical equivalence with DXA based on ±2% equivalence regions and strong linear relationships (Pearson’s *r* 0.90; concordance correlation coefficient 0.89–0.90). Across equations, mean absolute error and standard error of the estimate values were ~ 3.5% and ~ 4.2%, respectively. No proportional bias was observed.

**Conclusion:**

While continual advances are likely, smartphone-based 3D scanning may now be suitable for implementation for rapid and accessible body measurement in a variety of applications.

## 1 Introduction

Recent advances in digital anthropometry have highlighted the use of smartphone cameras to obtain visual information that can be used to produce 3-dimensional (3D) humanoid avatars. Several reports have supported the reliability of anthropometric and body composition parameters estimated by such procedures (1–4). While mobile digital anthropometry applications have typically constructed rigid humanoid avatars using two photographic images of static subjects—either from anterior and lateral (2–7) or anterior and posterior views (6, 8)—we recently reported the high reliability of new methods capturing serial images (~150) during complete rotation of subjects in front of a smartphone camera, followed by non-rigid avatar reconstruction (7). Specifically, the observed technical error of measurement (TEM) across common body circumferences averaged 0.5 cm or 0.9%, slightly lower than errors observed for two large, non-portable 3-dimensional scanning measurement booths that employ rigid avatar reconstruction and are commonly used in research and practice (TEMs of 0.6–0.8 cm or 1.1—1.5%). The combination of greater quantities of visual data and improved data processing pipelines may have contributed to these low errors.

In addition to establishing the reliability of body circumferences from smartphone 3D scanning, considering the validity of subsequent body composition estimation from humanoid avatars is warranted based on the importance of body composition in health, disease, and athletic settings (9–11). Trials to date have evaluated the validity of mobile applications estimating body composition variables from rigid avatars arising from two photographic images, with mixed results (2–6). These methods involve the assessment of a rigid, non-moving human body, which leads to relatively simple avatar reconstruction. Non-smartphone methods, such as traditional scanning booths or sensors positioned in front of turntables, have also employed rigid avatar reconstruction due to the lack of body movement during assessments. In contrast, emerging smartphone methods require participants to complete 360° of rotation in place by taking small, rocking steps while attempting to maintain an A-pose (i.e., standing upright with feet apart and legs straightened, arms straightened and lifted away from the sides of the body). Due to complex body motions generated during this rotation and the resultant body deformations, the 3D avatar must be produced using non-rigid reconstruction, potentially introducing additional error. Following both rigid and non-rigid avatar reconstruction, anthropometric variables from the avatars are used to predict body composition. However, no prior investigations have evaluated the validity of body composition estimates arising from smartphone-based scanning followed by non-rigid avatar reconstruction. Therefore, the purpose of the present study was to examine the validity of body fat percentage (BF%) prediction equations employed by such a smartphone-based 3D scanning application. It was hypothesized that BF% estimates obtained by the smartphone would exhibit strong linear relationships and statistical equivalence as compared to dual-energy X-ray absorptiometry (DXA), an accepted laboratory method of body composition assessment.

## 2 Method

### 2.1 Overview

Across two laboratories, adult participants were assessed using a smartphone 3D scanning application and dual-energy X-ray absorptiometry (DXA) at a single research visit. Serial images were collected by the smartphone 3D scanning application during a subject’s complete rotation in place, with data subsequently processed using non-rigid avatar reconstruction. The reliability of BF% from duplicate 3D scans was examined, and the validity of BF% values obtained by the 3D scanning application was established through comparison with DXA values.

### 2.2 Participants

Generally healthy adults (≥18 years of age) were recruited for participation in Lubbock, TX, USA and Baton Rouge, LA, USA. Prospective participants were ineligible if they had a diagnosis of a disease or any medical condition that is known to influence body composition (e.g., Cushing’s Syndrome, cancer, type 2 diabetes, chronic kidney disease, and heart failure), a history of major body altering surgery, implanted electrical devices, or were currently pregnant or breastfeeding. All participants provided written informed consent prior to participation, and this study was approved by the Texas Tech University Institutional Review Board (IRB2022-610; date of first approval: 07/23/2022) and the Pennington Biomedical Research Center Institutional Review Board (IRB 2022–002; date of first approval: 2/26/2022). All research was performed in accordance with relevant guidelines and regulations, including the Declaration of Helsinki.

### 2.3 Laboratory visit

Participants reported to the research laboratory at Texas Tech University (Lubbock, TX, USA) or Pennington Biomedical Research Center (Baton Rouge, LA, USA) after an overnight (≥8 h) period of fasting from foods, fluids, and other substances, and a ≥ 24-h abstention from exercise and other moderate- or vigorous-intensity physical activity. For assessments, each participant wore minimal form-fitting clothing.

### 2.4 Smartphone 3D scanning application

The smartphone 3D scanning application required participants to rotate in place on the laboratory flooring, using their own feet to perform the rotation and maintaining an A-pose, approximately 1.7 meters in front of a smartphone. During the rotation, multiple images were captured by the smartphone’s built-in camera. Scans were performed using an iPhone 13 Pro Max (model number MLKR3LL/A) with iOS v. 16.5 (Apple, Cupertino, CA, USA) or an iPhone 14 Pro (model number MQ2T3LL/A) with iOS v. 16.6. Each phone was mounted on a tripod for image acquisition. Each scan was automatically processed using the procedures of the manufacturer (Prism Labs, Los Angeles, CA, USA), which include machine learning for data pre-processing through binary segmentation and obtaining frame-to-frame correspondences (7). Humanoid avatars were produced by fully non-rigid reconstruction, and a parameterized body model was fitted to each avatar to normalize the avatar’s pose to a canonical pose and promote consistent measurement locations (1). Three scans were performed for each participant, and one scan was randomly selected for each participant, such that the present analysis is based on a single scan per participant to mimic typical use. For these scans, two proprietary BF% algorithms developed by the manufacturer were used: COmpound Circumferences Only (COCO) and Automatic Detection of Athlete Mode (ADAM). The COCO equation employs measurement ratios, such as waist:height, to estimate BF% using coefficients derived from linear regression on the manufacturer’s proprietary training data. The ADAM equation computes a weighted average between the COCO BF% and a variant of the Navy method designed to target individuals with lower BF%.

### 2.5 Dual-energy X-ray absorptiometry

A DXA scan was performed for each participant using a scanner that was calibrated daily according to manufacturer procedures (iDXA, General Electric, Boston, MA, USA with enCORE software versions 13.60.033 and 16.10.151, 16 [SP 1]). For each scan, the participant was positioned supine on the DXA table with hands neutral at their sides and feet together. Consistent positioning of hands and feet was achieved using foam blocks and straps. The region BF% values for the entire body were used in the present analysis.

### 2.6 Statistical analysis

The reliability of the ADAM and COCO 3D scanning equations was determined by calculating the TEM (i.e., precision error), least significant change (i.e., 2.77 × TEM), and the intraclass correlation coefficient (model 2.1) from duplicate scans, using previously described procedures (12, 13).

The validity of the ADAM and COCO 3D scanning equations were compared to reference DXA values. The linear relationships between 3DO and criterion estimates were established using ordinary least squares regression, with DXA specified as the *x* variable and the 3D scanning equation specified as the *y* variable. To determine if 3DO values demonstrated group-level statistical equivalence with DXA values, equivalence testing (14) was performed using equivalence regions of ±2.0% for BF%, as in a prior investigation (15). The mean difference (i.e., constant error) was calculated, along with the standard error of the estimate (SEE), root mean square error (RMSE), mean absolute error (MAE), Pearson’s *r* and R^2^, and Lin’s concordance correlation coefficient (CCC). Bland–Altman analysis was performed to establish the 95% limits of agreement, alongside linear regression to check for proportional bias (16). Statistical significance was accepted at *p* < 0.05. All statistical analyses were conducted in R (version 4.3.1) (17).

## 3 Results

### 3.1 Participants

**Figure 1:**
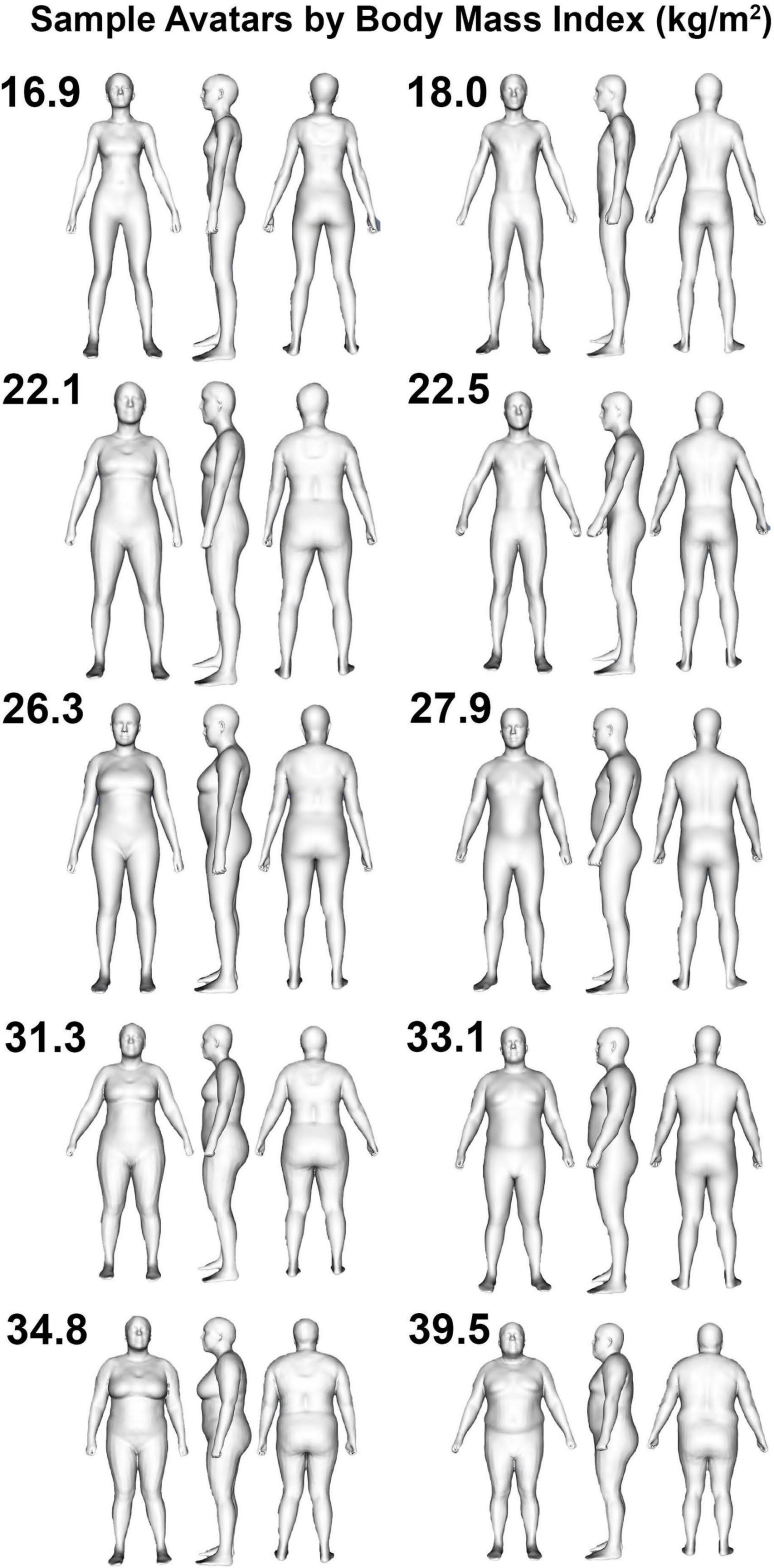
Humanoid avatars from 3-dimensional optical imaging scans. Sample female (left column) and male (right column) avatars are displayed for participants categorized as being underweight (i.e., BMI < 18.5 kg/m^2^; top row), healthy weight (i.e., 18.5 kg/m^2^ < BMI < 24.9 kg/m^2^; second row from top), overweight (i.e., 25.0 kg/m^2^ < BMI < 29.9 kg/m^2^; middle row), or obese (i.e., BMI > 30.0 kg/m^2^; bottom two rows).

One hundred and thirty-one participants (73 M, 58 F) with at least one valid scan were included in the validity analysis (Table 1), and a subset of 121 participants with two valid scans were included in the reliability analysis due to the need for duplicate scans to assess reliability. Sample avatars in differing body mass index categories are displayed in Figure 1. Based on self-report, 86 participants were non-Hispanic Caucasian, 21 were Hispanic Caucasian, 13 were Black or African American, 8 were Asian, 2 were Native American or Alaskan, and 1 was Native Hawaiian or other Pacific Islander.

**Table 1:** Participant characteristics.

	All (*n* = 131)				M (*n* = 73)				F (*n* = 58)			
	Mean	SD	Min	Max	Mean	SD	Min	Max	Mean	SD	Min	Max
Age (y)	33.7	16.0	18.0	76.0	36.2	16.8	18.0	76.0	30.5	14.5	18.0	72.0
Height (cm)	172.2	10.0	151.5	194.9	178.4	7.7	163.4	194.9	164.5	6.9	151.5	183.3
Weight (kg)	81.5	21.4	42.0	168.9	90.9	21.3	54.4	168.9	69.8	14.9	42.0	105.4
BMI (kg/m^2^)	27.3	5.9	16.9	48.5	28.5	6.0	17.8	48.5	25.8	5.5	16.9	41.9
DXA BF%	29.9	9.9	10.6	54.7	26.7	9.8	10.6	49.4	33.9	8.6	16.9	54.7

### 3.2 Reliability

For BF% from the ADAM equation, the TEM, least significant change, and ICC were 0.66%, 1.82%, and 0.996 (95% CI: 0.994–0.997), respectively. For BF% from the COCO equation, the TEM, least significant change, and ICC were 0.50%, 1.39%, and 0.997 (95% CI: 0.996–0.998).

### 3.3 Validity

**Figure 2:**
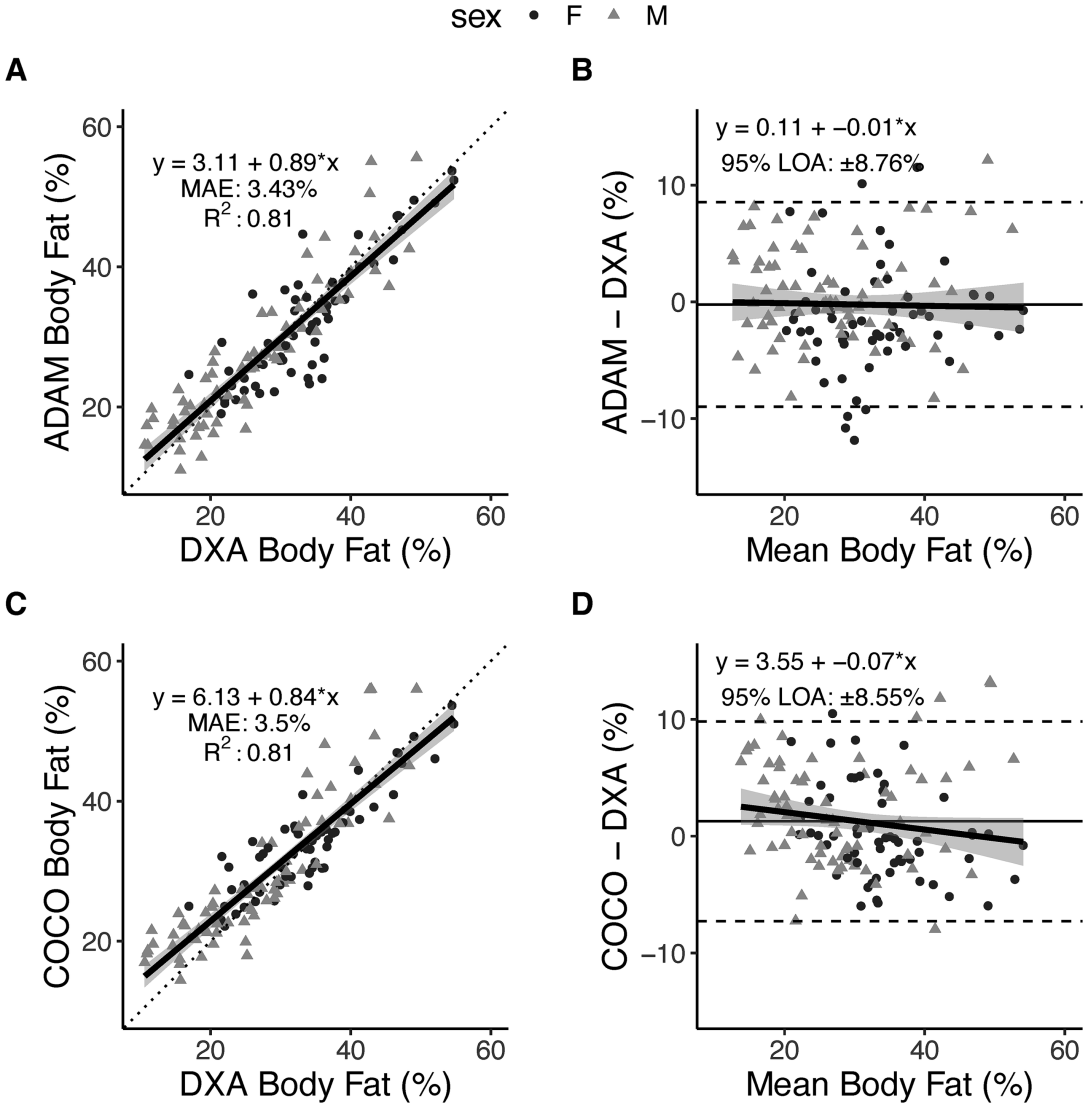
Body fat percentage estimates from smartphone-based 3-dimensional optical imaging. Two prediction equations, ADAM and COCO, were evaluated as compared to DXA. Relationships between 3DO-based values and DXA values are displayed in panels **(A,C)**, with the dotted line indicating the perfect linear relationship (line of identity) and the solid black line with shading representing the observed linear relationship and 95% confidence bands. Data points indicate individual participants. Bland–Altman plots are displayed in panels **(B,D)**, with dashed lines indicated the 95% limits of agreement, the horizontal solid black line indicating the mean difference (i.e., constant error), and the diagonal black line with shading indicating the observed linear relationship and 95% confidence bands.

Based on the prespecified equivalence regions of ±2.0%, both 3DO BF% equations (ADAM and COCO) demonstrated statistical equivalence DXA BF% (Table 2). Both equations also demonstrated strong, significant correlations with DXA (r 0.90; CCC 0.89–0.90; Figures 2A,C). MAE and RMSE values were 3.4–3.5 and 4.5%, respectively. From Bland–Altman analysis, no proportional bias was observed for either equation (ADAM equation: slope −0.01, 95% CI −0.09, –0.07, Figure 2B; COCO equation: slope −0.07, 95% CI −0.15, 0.01, Figure 2D). Limits of agreement ranged from 8.6 to 8.8%.

**Table 2:** Validity results.

DXA				3D scanning					Validity analysis						
Mean	SD	Min	Max	BF% estimate	Mean	SD	Min	Max	MD	SD of MD	SEE	RMSE	*r*	CCC	Equivalence?
29.9	9.9	10.6	54.7	ADAM	29.7	9.8	11.0	55.6	−0.2	4.5	4.3	4.5	0.90*	0.90*	Y (*p* < 0.01)
				COCO	31.1	9.3	14.4	56.0	1.3	4.4	4.1	4.5	0.90*	0.89*	Y (*p* = 0.03)

MD, mean difference; SEE, standard error of the estimate; RMSE, root mean square error; r, Pearson’s correlation coefficient; CCC, Lin’s concordance correlation coefficient; Y, yes (statistically equivalent); BF%, body fat %; ADAM, Automatic Detection of Athlete Mode 3D scanning equation; COCO, COmpound Circumferences Only 3D scanning equation.

*p < 0.001.

## 4 Discussion

Smartphone-based 3D scanning increases the accessibility of digital anthropometry and body composition estimation. While such mobile scanning methods have typically relied on the generation of rigid avatars from two photographic images, new methods employ the acquisition of numerous images to capture more body shape data for use in non-rigid avatar reconstruction. With recent data indicating the precision of anthropometric and body composition estimates from this method compares favorably to traditional, non-portable 3D scanners (7), a consideration of the validity of resultant body composition estimates was warranted. In the present analysis, we found that two prediction equations demonstrated high reliability and generally strong agreement with DXA for estimation of BF%.

For both BF% equations, very high reliability was observed, with TEM values of 0.50–0.66% from duplicate assessments. Corresponding least significant change values, reflecting the degree of change that would be considered statistically significant, were 1.39–1.82%. Additionally, strong group-level agreement was observed, as supported by statistical equivalence with DXA and strong linear relationships (*r* 0.90; CCC 0.89–0.90). Several additional metrics (SEE, RMSE, and MAE) described the typical individual errors of the equations, with values ranging from 3.4 to 4.5% across metrics and equations. Bland–Altman analysis did not indicate proportional bias in either equation, which is an encouraging indicator due to the common occurrence of large negative proportional bias when applying body composition prediction equations, particularly in consumer-facing assessment methods (15, 18). For example, we previously found notable proportional bias, with slopes of −0.27 to −0.35, when evaluating anthropometric BF% prediction equations developed using the NHANES dataset (15). Additionally, in an evaluation of numerous consumer-grade bioimpedance scales, we found that approximately half exhibited notable proportional bias for BF%, with slopes as large as −0.50 (18). Despite the minimal proportional bias in the present study, the limits of agreement were approximately ±8.6% for both equations, indicating a relatively wide range of individual-level differences between DXA and the prediction equations are possible. However, typical errors—as indicated by the SEE—may be closer to ≤ ±4% in two-thirds of cases. Collectively, these results support high reliability and group-level performance of the prediction equations and provide information regarding the individual-level errors that can be expected with this technology.

A small number of previous investigations have reported the validity of smartphone-based 3D scanning applications, typically using two photographic images, as compared to reference methods (2, 6, 8). Graybeal et al. (2) demonstrated a similar high reliability of BF% estimates (TEM of 0.3–0.4%) and good group-level performance as compared to a rapid 4-compartment model (*r* 0.85; statistical equivalence between methods based on a ± 2% equivalence region). However, RMSE values (5.0–5.1%) were slightly higher than in the present investigation (4.5%), and a larger magnitude of proportional bias was observed (slope of −0.25 vs. −0.01 to −0.07 in the present study). In a separate investigation using different 3D scanning applications, Graybeal et al. (6) observed TEM values of 0.3–0.6% for BF%, RMSE values of 3.9–6.2%, and statistical equivalence for some, but not all, scanning applications. As in other studies, negative proportional bias was observed, with slopes of −0.17 to −0.53 across applications. Collectively, some aspects of the performance of the 3D scanning applications evaluated in the present study are similar to prior investigations, with the reduction in the magnitude of proportional bias being a potentially notable difference.

The participants in the present investigation comprised a wide range of adiposity, with DXA BF% values of 10.6–54.7% and BMIs of 16.9–48.5 kg/m^2^, as well as expected natural variation in overall body size and shape. An approximately even distribution between sexes (73 M, 58 F) and some representation of racial or ethnic minorities (35% of the sample) were also features of the sample. Collectively, these features contributed to a relatively diverse sample in terms of body size and composition, race and ethnicity, and sex. However, a limitation is the relatively young average age (33.7 ± 16.0 years). As such, the present results provide an important step in evaluating the smartphone-based 3D scanning procedures, but continued investigation is warranted in a variety of groups, including diverse racial and ethnic groups and middle-aged or older adults.

Smartphones are ubiquitous worldwide, with 2022 estimates indicating a median adult smartphone ownership rate of 85% across 18 advanced economies—an increase from 76% in 2018 (19, 20). As such, numerous promising applications of smartphone-based health technologies can be considered. The accessibility of smartphone-based 3D scanning allows for precise anthropometric evaluation and subsequent body composition estimation, providing new opportunities for individual users to track relevant body changes over time. For example, a simple implementation of this technology is the ability for smartphone-based 3D scanning to provide a precise estimate of waist circumference, thereby allowing one important component of cardiometabolic risk (21) to be easily assessed without the need for a trained assessor. Additionally, there are opportunities for anthropometric and body composition estimates to be integrated into weight management mobile applications to provide customized feedback and progress tracking. While the ability of 3D scanning to aid in the success of such weight management programs will be a topic for future investigation, the automated nature of such procedures reduces barriers to physical evaluations as compared to decades past. The ability to rapidly obtain automated measurements at home, using smartphone capabilities, could eliminate the need for in-person anthropometric assessment by health providers. Beyond using simple metrics like waist circumference and BF%, there are also opportunities to employ various machine learning and artificial intelligence procedures to characterize unique body phenotypes and their relationship to health and disease parameters (22, 23). Pairing smartphone-based 3D scans with relevant clinical data—such as blood lipids, glucose, and blood pressure—may allow for better understanding of the influence of body shape and size on relevant cardiometabolic risk factors, both at the group and individual level. Future investigations including a greater proportion of participants with obesity and related comorbidities will provide further clarity regarding the utility of this technology. Due to the lack of risk and non-invasive nature of 3D scanning assessments, other medical applications—such as the monitoring of pregnant and breastfeeding individuals—should also be considered in subsequent work. While future research and development will be needed to realize the potential of 3D scanning as a component of health assessment, emerging findings indicate notable potential of smartphone-based methods.

In summary, the present study demonstrates the validity of body composition estimation from smartphone-based 3D scanning. Unlike previous trials of smartphone technologies, the humanoid avatars constructed by the 3D scanning application were based on large amounts of visual data collected during complete subject rotation. With the reliability (7) and validity of these procedures established, new applications of this technology can be investigated. Additionally, continued refinement of body composition prediction in diverse populations can promote the lowest errors achievable and maximize the ability to accurately track changes over time. While continual advances are likely, smartphone-based 3D scanning may now be suitable for implementation for rapid and accessible body measurement in a variety of applications.

## Data Availability

The datasets presented in this article are not readily available because institutional approval is required. Requests to access the datasets should be directed to grant.tinsley@ttu.edu.
